# More Novel Hantaviruses and Diversifying Reservoir Hosts — Time for Development of Reservoir-Derived Cell Culture Models?

**DOI:** 10.3390/v6030951

**Published:** 2014-02-26

**Authors:** Isabella Eckerle, Matthias Lenk, Rainer G. Ulrich

**Affiliations:** 1 Institute of Virology, University of Bonn Medical Centre, Sigmund-Freud-Strasse 25, 53127 Bonn, Germany; 2 Department of Experimental Animal Facilities and Biorisk Management, Friedrich-Loeffler-Institut, Federal Research Institute for Animal Health, Südufer 10, 17493 Greifswald-Insel Riems, Germany; E-Mail: Matthias.Lenk@fli.bund.de; 3 Institute for Novel and Emerging Infectious Diseases, Friedrich-Loeffler-Institut, Federal Research Institute for Animal Health, Südufer 10, 17493 Greifswald-Insel Riems, Germany; E-Mail: rainer.ulrich@fli.bund.de

**Keywords:** hantavirus, cell culture, zoonoses, reservoir host, virus-host interaction

## Abstract

Due to novel, improved and high-throughput detection methods, there is a plethora of newly identified viruses within the genus *Hantavirus*. Furthermore, reservoir host species are increasingly recognized besides representatives of the order Rodentia, now including members of the mammalian orders Soricomorpha/Eulipotyphla and Chiroptera. Despite the great interest created by emerging zoonotic viruses, there is still a gross lack of *in vitro* models, which reflect the exclusive host adaptation of most zoonotic viruses. The usually narrow host range and genetic diversity of hantaviruses make them an exciting candidate for studying virus-host interactions on a cellular level. To do so, well-characterized reservoir cell lines covering a wide range of bat, insectivore and rodent species are essential. Most currently available cell culture models display a heterologous virus-host relationship and are therefore only of limited value. Here, we review the recently established approaches to generate reservoir-derived cell culture models for the *in vitro* study of virus-host interactions. These successfully used model systems almost exclusively originate from bats and bat-borne viruses other than hantaviruses. Therefore we propose a parallel approach for research on rodent- and insectivore-borne hantaviruses, taking the generation of novel rodent and insectivore cell lines from wildlife species into account. These cell lines would be also valuable for studies on further rodent-borne viruses, such as orthopox- and arenaviruses.

## 1. Introduction

Emerging zoonotic viruses have received tremendous interest within recent years and are perceived as a major health risk for humans [1,2]. Among them are many RNA viruses from wildlife reservoirs, with recent examples including Severe Acute Respiratory Syndrome (SARS) and Middle East Respiratory Syndrome (MERS) Coronavirus (CoV) as well as Old- and New World hantaviruses [3,4,5,6,7,8,9,10]. In addition, recent “pathogen hunting” approaches resulted in the discovery of novel paramyxo-, hepe-, hepaci- and hepadnaviruses in bats and rodents [11,12,13,14,15,16,17].

Hantaviruses are enveloped viruses with a segmented RNA genome of negative polarity. Taxonomically, these viruses belong to the genus *Hantavirus* within the family *Bunyaviridae* [18]. In contrast to other genera of the family *Bunyaviridae*, hantaviruses are harbored by small mammals, mainly rodents (order Rodentia). In general, each hantavirus species is thought to be carried and transmitted to humans by a single reservoir species. Thus, the prototype hantavirus species, the Hantaan virus, was exclusively detected in the striped field mouse *Apodemus agrarius* in Asia [19]. Similarly, the European Puumala virus (PUUV), causing the majority of human infections in Northern, Western and Central Europe, seems to be adapted to the bank vole *Myodes* (*Clethrionomys*) *glareolus* [20,21]. For other rodent-borne hantaviruses, such as Dobrava-Belgrade virus, different genotypes have been identified, each harbored preferentially by a defined *Apodemus* species [22]. In addition, Tula virus (TULV) was initially detected in the common vole *Microtus arvalis* representing the reservoir host, but was also molecularly detected in other vole species [23,24,25]. The multiple detection of TULV in different putative reservoirs underlines the problems of the identification of reservoir hosts and the necessity of comprehensive field studies in habitats with sympatrically occurring putative reservoir species [24,26].

Recently, a large number of shrews and moles (order Soricomorpha/Eulipotyphla) and bats (order Chiroptera) have been identified as reservoirs of additional hantaviruses [21,27,28,29,30,31,32]. The discovery of these highly divergent hantaviruses challenge the previous assumption of a strict virus-host coevolution over long time scales. Besides the virus-host coevolution hypothesis, alternative scenarios of host-switch events and adaptive evolution have been discussed recently [33,34,35,36,37,38]. Finally, recent findings of spillover infections of European hantaviruses, such as TULV and Dobrava-Belgrade virus, raise important questions on the host range of hantaviruses and the determinants of host specificity (see e.g., [24,39]). In humans, hantaviruses are known to cause a broad range of clinical disease, mainly affecting the renal tract: hemorrhagic fever with renal syndrome (HFRS), with its milder form, nephropathia epidemica (NE), or the respiratory tract: hantavirus cardiopulmonary syndrome (HCPS) caused by certain Old and New World hantaviruses, respectively [10,21,40]. Similarities in the clinical pictures of both syndromes and overlap of clinical presentation such as pulmonary symptoms in a subset of patients with NE and kidney involvement in some patients with HCPS suggest that the previously used dichotomy of clinical presentation might be not useful to describe the clinical outcome of human hantavirus infections and to understand its underlying pathological processes [41,42,43,44,45,46]. Therefore, the term “hantavirus disease” was proposed [47].

In contrast, in the reservoir host a hantavirus infection is usually believed to be persistent and not associated with (at least obvious) disease. However, field studies in bank voles indicated that hantavirus infection has a negative impact on over-winter survival, and histopathologic lesions in several organs have been observed in hantavirus-infected white-footed mice and in deer mice [48,49,50]. The causal relationship and the relevance of these observations remain, however, unknown.

With the discovery of hantaviruses in a broad spectrum of reservoirs and the identification of unexpected spillover and putative host-switch events, reservoir-host centered infection models become of immediate interest [10,51]. In addition, public health-related questions on the potential of these viruses to overcome species barriers and a rational-based risk assessment arise. This assessment is not only restricted to the field of hantavirus research but relevant in the context of many emerging virus species, such as members of the families *Corona*- or *Paramyxoviridae.* These research questions necessitate, however, novel tools and methods that allow comparative infection studies and immunological evaluation of virus-host interactions among a broad species context, not only including wildlife, but also livestock and companion animal species.

Until now, there is little knowledge on virus-host interaction in the natural reservoir; and currently available *in vitro* model systems do not reflect characteristics of reservoir specific virus-host interactions [52]. Animal infection experiments with zoonotic viruses under laboratory conditions in the natural reservoir hosts are limited to a small number of species and to highly specialized laboratories. Currently available *in vivo* data on the immunology of hantavirus infection in their natural reservoir stem from animal experiments on Norway rats, deer mice, bank voles and cotton rats (for an overview see [53]). However, reservoir animal models for *in vivo* studies are not available for the majority of species that harbor hantaviruses. The main reasons are difficulties in breeding and keeping these species under laboratory conditions. Furthermore, many rodents as well as members of the orders Soricomorpha/Eulipotyphla and Chiroptera are protected species; therefore they are not available in large numbers for animal experiments or for *in vivo* studies in the laboratory at all. An overview on model systems for the study of zoonotic viruses is presented in Table 1.

Among all mammalian reservoir hosts, bats are most likely the order of mammals that has received the most attention from the virological research community in recent years [54,55,56]. A plethora of novel viruses have been identified in bats, among them many zoonotic viruses. However, most bat species, similar to rodents and insectivores, are not available for animal experiments, and this has stimulated the establishment of novel *in vitro* models such as bat cell lines. The generation of these cell lines across a broad range of species has already provided important insights into virus-host interaction on a cellular level and on innate immune functions, e.g., interferon response [57,58,59,60,61,62,63].

The many similarities between bat-, insectivore- and rodent-borne viruses could stimulate a synergistic approach for reservoir host-derived *in vitro* models to study hantaviruses.

**Table 1:** Overview of model systems for the study of zoonotic viruses.

Model	Advantages	Disadvantages	Value for zoonosis research
Animal model, conventional (e.g., laboratory mouse, rat)	Easy to maintain and breed	Heterologous pathogen-host relationship	Limited
	Species-specific reagents available	Transfer of results to humans or reservoir host limited	
Animal model, reservoir host (natural reservoir host species)	Homologous pathogen-host relationship	Husbandry and breeding limited to few species	High, but limited to few species
		Species-specific reagents rarely available	
Cell culture, conventional (e.g., Vero E6, tumor cell lines, HUVECs, monocytes, dendritic cells)	Easy to culture	Heterologous pathogen-host relationship	Suitable as a basic model, but less useful for more complex questions on pathogen-host interaction
	Assays, reagents and methods available	Accumulation of mutations/deregulation of important cellular pathways due to high passage numbers possible	
Cell culture, reservoir host-derived	Homologous pathogen-host relationship	Very few reservoir-derived cell lines available so far	High
		Thorough characterization necessary	
		Species-specific reagents rarely available	

## 2. What Have We Learned from Reservoir-Derived Cell Lines in the Field of Bat-Borne Viruses?

### 2.1. Virus Isolation Studies

Cell culture is the mainstay of classical virology and even in times of highly sensitive and high-throughput detection methods, isolation of a virus in cell culture enables its thorough phenotypical characterization. While in the early era of virology, isolation by cell culture was routinely performed; it has become something of an outdated method for many years. Cell lines such as Vero cells, derived from the kidney of an African green monkey, are widely used for virus isolation and especially the subclone Vero E6 provides an excellent environment for RNA viruses to replicate due to an impaired IFN response [64,65].

Vero E6 cells have enabled the isolation of a variety of hantaviruses [29,66,67,68,69,70], but most novel identified bat-, insectivore- and rodent-borne viruses remain uncultured. Therefore, primary and immortalized cell lines derived from reservoir hosts could provide a benefit for virus isolation. Several examples provide evidence that reservoir-derived cells can be beneficial over conventional cell lines to isolate reservoir-borne viruses: The henipa-related paramyxovirus Cedar virus was firstly isolated in primary kidney cells derived from a flying fox (*Pteropus alecto*), the species which naturally harbors this virus [71]. In this study, only primary bat cells showed a cytopathic effect while several other cell lines, including Vero cells, did not. This cell line was also used for successful isolation of Menangle virus, another zoonotic paramyxovirus [72]. In addition, Zhang *et al.* showed isolation and replication of a bat herpesvirus derived from the bat *Miniopterus schreibersii* in primary bat cells after unsuccessful isolation attempts in 14 other mammalian cell lines [73].

Although hantaviruses are not as hard to isolate as other zoonotic viruses (*i.e.*, members of the family *Coronaviridae*), the number of hantavirus isolates of reservoir or human origin is still rather small [74]. Therefore, the use of reservoir-derived cell lines might be also beneficial for the generation of a more comprehensive collection of hantavirus isolates. 

### 2.2. Virus Evolution and Adaptation during Cell Culture Propagation

Many hantavirus isolates have been obtained a long time ago and were propagated on conventional cell lines such as Vero E6 cells. Therefore, they might have accumulated adaptations to the cell lines on which they have been propagated and do not fully display all characteristics of the viruses found in the reservoir. One example that supports this consideration is the report of the attenuation of a PUUV strain which subsequently lost its ability to infect the natural reservoir animal after passaging due to the accumulation of mutations in the S segment. Here, a wild-type variant that was passaged in bank voles was well adapted for reproduction in the reservoir host but not in cell culture, while the strains propagated on Vero E6 replicated to much higher efficiency in cell culture but did not reproducibly infect bank voles [75]. It was further observed that hantavirus strains evolve during multiple cell culture passages. For example, Sundström *et al.* isolated PUUV strains which differed from the corresponding parental strain by plaque size, the ability to replicate in interferon-defective *versus* interferon-competent cell lines and the potential to induce innate immune responses [76].

The emergence of a stop codon within the coding sequence of the NSs open reading frame of TULV may represent also an adaptation of the virus to the IFN-deficient Vero E6 cell line [77]. Similarly, the PUUV prototype strain passaged in Vero E6 cells was demonstrated to contain two sequence variants, an NSs-intact variant and a stop codon containing NSs variant [78]. In contrast, the vole reservoir-derived PUUV and TULV strains were found to contain a conserved intact NSs open reading frame which might be functional in the reservoir [79].

### 2.3. Deciphering Replicative Capacity of Reservoir-Associated Viruses in Reservoir-Host Cell Lines

Reservoir-derived cell lines are not only a suitable tool for the study of evolutionary closely linked virus-host combinations, but they can also be used for deciphering cross-species transmission, hinting at a certain species as animal reservoir or estimating replicative capacity. Examples include assessment of the replicative capacity of the newly emerged MERS-CoV in bat cell lines not only originating from the presumed reservoir host, bats of the family Vespertilionidae, but across several other bat families and ungulates [80,81] and identification of the MERS-CoV receptor [82]. Further, New World bat and cotton rat-derived cell lines were successfully used for the characterization of a sylvatic isolate of St. Louis encephalitis virus [83]. Bat cell lines obtained from *Pipistrellus ceylonicus* allowed propagation of a rhabdovirus pathogenic to humans, Chandipura virus, and a bat adenovirus isolated from *Rousettus leschenaulti* from India, while the cells did not support replication of a number of bunya-, alpha- and flaviviruses [84]. As bats have recently been identified as hosts of influenza viruses [85,86], bat cells were shown to be susceptible to influenza A virus infection, and importantly, to allow re-assortment during co-infection of two influenza viruses [87].

On the other hand, reservoir-derived cell lines from bats could provide hints to a wildlife origin of human viruses that are already circulating in the human population for some time: For example, Huynh and coworkers showed replication of the human coronavirus HCoV-NL63 in immortalized lung cells from the North American tri-colored bat (*Perimyotis subflavus*) for multiple passages, suggesting an origin of the virus in bats [88].

### 2.4. *In Vitro* Studies on Virus-Host Interaction

A main application of reservoir-derived cell lines is the investigation of virus-host interactions upon controlled infection experiments. Bat cell lines have already served as a valuable tool to study virus entry and replication among a broad range of zoonotic viruses in models representing the natural reservoir host. For example, two filoviruses, Ebola virus (EBOV) and Marburg virus which are harbored by bats in the wild, were shown to enter and replicate efficiently in a bat cell line derived from the Egyptian fruit bat (*Rousettus aegyptiacus*), indicating that this model is highly suitable to investigate the biology of filoviruses in cells derived from their presumed reservoir [89]. Furthermore, it has been shown that the glycoprotein of EBOV can interact in fruit bat and human cells in a similar manner and does not limit EBOV tropism to certain bat species [90]. Further, the glycoprotein of Lloviu virus, a filovirus from bats that has not been isolated so far, was found to mediate cellular entry in similar manner to other filoviruses with a tropism for bat cells derived from multiple species [91]. Surface glycoproteins of African henipaviruses could induce syncytium formation in a cell line derived from an African fruit bat, indicating a similar strategy of virus entry for both Asian and African henipaviruses, and providing a cell culture model for isolation of these emerging viruses [92].

Besides entry studies, reservoir-derived cell lines from bats could also provide insights into the reservoir host innate immune response to paramyxoviruses. By the use of reservoir-derived cell lines, it was shown that interferon production and signaling pathways are antagonized during henipavirus infection of fruit bat cell lines [58]. Further insight into the interferon system of bats was gained by characterization of the type I interferon reaction to viral infection in interferon-competent, immortalized cell lines from the African fruit bat *Eidolon helvum* [57].

The above-mentioned differences in the host range of hantaviruses might be driven by polymorphisms in the receptor molecules. Thus, closely related *Microtus* species (*M. arvalis*, *M. agrestis*) may have a similar hantavirus entry receptor, although they are phylogenetically a long time separated as reflected in their morphological features [24]. In addition, the tissue tropism of hantaviruses in their natural reservoirs might be determined by the receptor repertoire, cellular cofactors of virus replication and transcription, and innate immunity mechanisms. Recently, it has been shown that pathogenic hantaviruses in contrast to non-pathogenic hantaviruses display a different induction of microRNAs, essential regulators of host immune response genes, in human endothelial cells, macrophages and epithelial cells [93]. To study these immunological regulators in the reservoir host as well would be an interesting option. Therefore, bat-, insectivore- and rodent-derived cell lines would represent a valuable tool for identification of host factors. Moreover, such cell lines would help to understand innate immunity escape mechanisms that are linked to the activity of the putative NSs protein, exclusively identified in arvicoline, sigmodontine- and neotomine-associated hantaviruses [34].

## 3. What Are the Obstacles to Overcome? — A Research Agenda for Reservoir-Derived Rodent and Insectivore Cell Lines

It has been shown that hantaviruses can infect multiple cell types, but have a tropism to endothelial cells in human infection, a reason why human umbilical vein endothelial cells (HUVECs) are the main cell culture model for hantavirus disease in humans [94]. Further, hantaviruses can infect dendritic cells which are suggested to significantly contribute to hantavirus pathogenesis in humans [95]. In rodents, which shed the virus in saliva, urine and feces, the highest amount of hantaviral RNA is consistently found in the lungs [96,97,98]. As transmission of hantaviruses between rodents and also during zoonotic transmission from rodents to humans is mainly through the respiratory route, the lung is of interest for hantavirus infection. Indeed, we could observe PUUV hantavirus infection in human primary airway epithelial cells as well as in an airway epithelial cell line derived from a bank vole [99] Further, as the viruses are shed in the urine, renal epithelial cells could be of interest for the study of virus-host interactions in the natural reservoir host. One of the few reservoir-derived cell lines that are already available from a hantavirus reservoir host is a spontaneously immortalized cell line derived from the kidney of an adult bank vole [100]. Although this cell line was permissive to several arthropod- and rodent-borne viruses such as Vesicular stomatitis virus, vaccinia virus, cowpox virus, Sindbis virus, Pixuna virus, Usutu virus, Inkoo virus, and Borna disease virus, it failed to allow productive infection with PUUV strain Vranica, a strain that is adapted to and passaged in Vero E6 cells. Another group isolated bank vole embryonic fibroblasts and showed that these primary cells were susceptible for PUUV-infection, including a wild‑type PUUV strain that was only passaged in bank voles [101]. However, this approach is dependent on the availability of embryonic organ material of bank voles, which requires a successful bank vole breeding colony to continuously obtain embryos for the preparation of primary cells. In case of the existence of a breeding colony of a given rodent or insectivore species, this approach might be especially useful for studies on selected cell types, e.g., dendritic cells, but might be not a general option for most hantavirus-harboring species.

To ensure the continuous availability of a cell culture model system, immortalization is an option to create infinite cell lines from primary cells. While in primary rodent cells spontaneous immortalization can occur, it is not known if bat or insectivore cells behave in the same way. Other methods of immortalization include retroviral systems such as lentiviral transduction of the coding sequence of large T antigen of SV40 or introduction and stable expression of telomerase reverse transcriptase protein, both attempts which have been already successfully applied for the generation of bat cell lines [57,81,102]. For an overview of advantages and disadvantages of primary *vs.* spontaneously *vs.* artificially immortalized cells, see Table 2.

To obtain suitable tissue of reservoir hosts, ongoing research projects on small mammals can be of use for collection of organ material to isolate primary cells. While it is not possible to generate cell lines from the plethora of rodent and insectivore species in which hantaviruses are found, a pragmatic approach is to focus on representative species. Selection criteria for these representative species could be: the overall importance of the associated virus, availability of breeding colonies, and representation of certain families/genera.

**Table 2:** Advantages and disadvantages of primary *vs.* spontaneously *vs.* artificially immortalized cell culture.

Cell characteristics	Primary cells	Spontaneously immortalized cells	Artificially immortalized cells
**Immortalization**	None	Occurs only after multiple passages	Possible at low passage number
**Genetic background**	Close to *in vivo* situation	Artificial (severe changes can occur upon immortalization)	Artificial (severe changes can occur upon immortalization)
**Proliferative capacity**	Mostly low *	High	High
**Life span**	Limited, finite	Not limited, infinite	Not limited, infinite
**Biological properties**	Close to *in vivo* situation	Dedifferentiation, more distant from *in vivo* situation	Dedifferentiation depends on immortalization methods
			Selection of distinct cell types possible, conditional immortalization strategy feasible
		Selection of distinct cell types difficult	
**Preferred culture medium **	Specialized medium (expensive) **	Standard medium	Standard medium
**Properties of cell population**	Close to *in vivo* cell types	Loss of distinct cell types during subcultivation	Selection of distinct cell types possible
**Standardization and reproducibility**	Limited cell number, standardization only possible for some cell types ***	Standardization possible due to unlimited amount of cells	Standardization possible due to unlimited amount of cells

* Exceptions of primary cells that have a high proliferative capacity are primary fibroblasts and HUVECs. They are, however, limited in their passage number. ** Exceptions include primary fibroblasts and HUVECs for which affordable media are widely available. *** Standardization of primary cells is possible to some extent given the case that cells of a low passage number can be sufficiently expanded after initial isolation, *i.e.*, HUVECs.

Natural reservoir host species that are available as laboratory animals, in breeding colonies or in enclosures (such as Norway rats, deer mice, bank voles, common voles, cotton rats or Asian house shrews) are of special interest: cell lines derived from these animals could be compared to the *in vivo* infection in an animal model with the associated virus species. Further, laboratory-bred populations are less likely to be infected with unwanted pathogens, and in most instances, stem from a genetically characterized background. 

**Figure 1:**
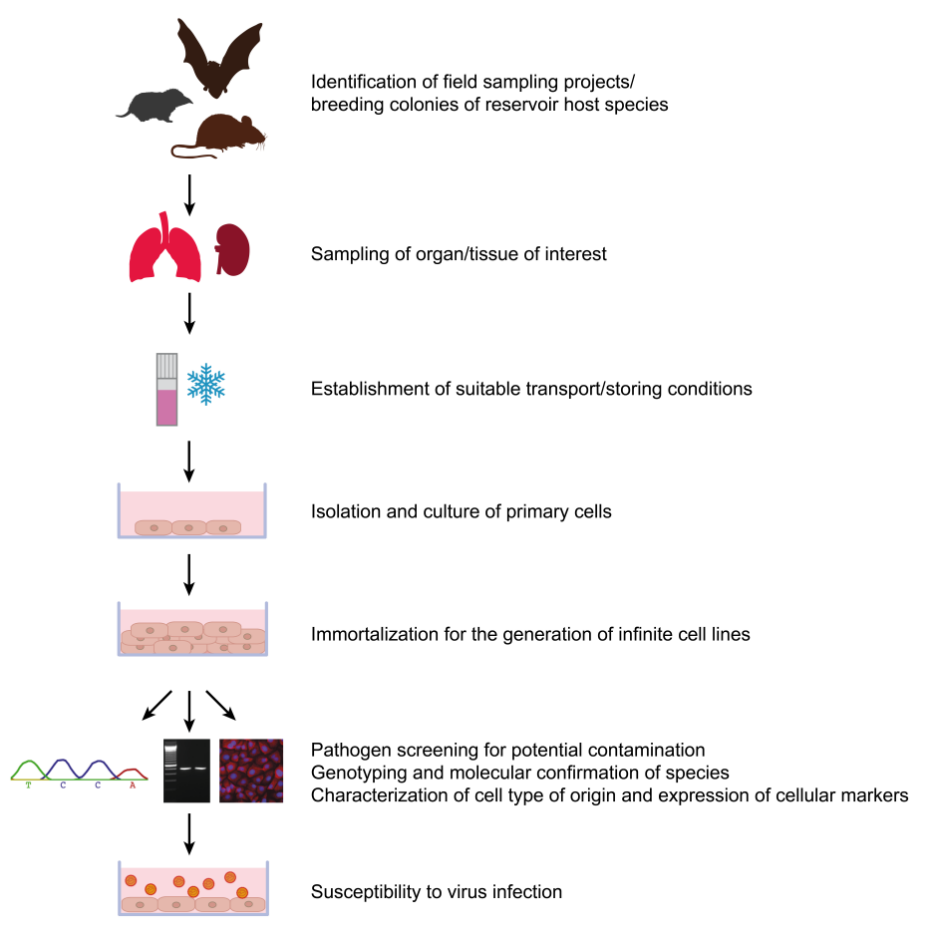
Algorithm for the generation of reservoir-derived cell lines from bats, insectivores and rodents. A similar approach has already been successfully applied for the establishment of bat, rodent and insectivore cell lines [57,80,81,83,103,104].

If cell lines are generated from feral animals, a thorough screening for contaminants from the field is necessary (contaminants include bacteria and parasites such as mycoplasma or trypanosomes as well as viruses that might be cultured along with primary cells). Animals from which tissue material was obtained should be either directly tested for contamination with known pathogens or alternatively sentinel animals, *i.e.*, laboratory mice are an option. Thorough screening, for example by next generation sequencing of organ material, can provide further assessment to ensure high-quality cell lines without contaminants, even for as yet unknown pathogens. Furthermore, thorough characterization of the cell lines is obligatory for their use in virus infection studies. This characterization should focus on the following questions: Do these cells still express relevant receptors, are interferon signaling pathways still intact, do these cells still represent important characteristics of their cell type of origin (*i.e.*, in the case of epithelial cells: are cell-cell contacts intact, do cells still form a monolayer, do they retain their ability to form a polarized monolayer?). Although complex, this characterization ensures a valid cell culture model, which can then easily be shared between different research groups and lead to novel insights into the highly conserved hantavirus-host interaction in the context of their natural reservoir. As seen in the field of bat-borne zoonoses, reservoir-derived cell lines can serve as a valuable *in vitro* tool and therefore this approach should also be used in the field of rodent- and insectivore-borne zoonotic viruses. Furthermore, synergistic approaches of bat-, rodent- and insectivore-borne viruses and their reservoirs might enable the identification of general mechanisms of virus persistence, conserved across a broad range of mammalian reservoir hosts. Protocols for the generation of bat cell lines have been established and could be adapted to the rodent and insectivore host [57,81,102]; an exemplary approach is presented in Figure 1. In order to mimic the natural infection as close as possible, generation of cell lines should focus on cell types which are a target during natural infection or stem from organs that are involved in virus entry, spread or shedding, such as epithelial cells from the respiratory or renal tract.

## 4. Conclusions

In conclusion, investing in a large range of reservoir-derived cell culture models will be a promising tool to reveal novel aspects of the hantavirus-host relationship. Further, experience and model systems from the field of bat-borne zoonosis can serve as a blueprint for the hantavirus research community. 
